# Interictal intracranial electroencephalography for predicting surgical success: The importance of space and time

**DOI:** 10.1111/epi.16580

**Published:** 2020-06-26

**Authors:** Yujiang Wang, Nishant Sinha, Gabrielle M. Schroeder, Sriharsha Ramaraju, Andrew W. McEvoy, Anna Miserocchi, Jane de Tisi, Fahmida A. Chowdhury, Beate Diehl, John S. Duncan, Peter N. Taylor

**Affiliations:** 1 CNNP lab (https://www.cnnp-lab.com), Interdisciplinary Complex Systems Group, School of Computing, Newcastle University, Newcastle Upon Tyne, UK; 2 Faculty of Medical Sciences, Newcastle University, Newcastle Upon Tyne, UK; 3 Institute of Neurology, University College London, London, UK

**Keywords:** cortical localization, EEG, epilepsy surgery, epileptogenic zone, intracranial electrodes

## Abstract

**Objective:**

Predicting postoperative seizure freedom using functional correlation networks derived from interictal intracranial electroencephalography (EEG) has shown some success. However, there are important challenges to consider: (1) electrodes physically closer to each other naturally tend to be more correlated, causing a spatial bias; (2) implantation location and number of electrodes differ between patients, making cross-subject comparisons difficult; and (3) functional correlation networks can vary over time but are currently assumed to be static.

**Methods:**

In this study, we address these three challenges using intracranial EEG data from 55 patients with intractable focal epilepsy. Patients additionally underwent preoperative magnetic resonance imaging (MRI), intraoperative computed tomography, and postoperative MRI, allowing accurate localization of electrodes and delineation of the removed tissue.

**Results:**

We show that normalizing for spatial proximity between nearby electrodes improves prediction of postsurgery seizure outcomes. Moreover, patients with more extensive electrode coverage were more likely to have their outcome predicted correctly (area under the receiver operating characteristic curve > 0.9, *P* « 0.05) but not necessarily more likely to have a better outcome. Finally, our predictions are robust regardless of the time segment analyzed.

**Significance:**

Future studies should account for the spatial proximity of electrodes in functional network construction to improve prediction of postsurgical seizure outcomes. Greater coverage of both removed and spared tissue allows for predictions with higher accuracy.

## 1 Introduction

Surgery is an effective treatment for epilepsy, with more than half of patients achieving outcomes of postoperative seizure freedom.^1^ For patients not seizure-free after surgery, a possible explanation is the incomplete removal of the epileptogenic zone, defined as the area of cortex that is indispensable for seizure generation.^2^ More recently, the concept of the epileptogenic network has emerged, recognizing multiple brain regions and connections between them to be responsible for generating seizures.^3,4^ Identification of the epileptogenic network in each patient is extremely challenging, as removal of multiple brain regions and connections between them may lead to seizure freedom. Several recent studies have highlighted network properties that are potentially indicative of the epileptogenic network and used various network properties to predict postoperative outcome.^5–9^

Functional networks inferred using intracranial electroencephalography (iEEG) have received considerable attention in this context. These functional networks use iEEG signal similarity as a measure of connection strength between iEEG channels. Studies using iEEG-derived networks have demonstrated their value for predicting patient outcomes when using ictal^10–14^ and interictal data.^15–20^

The potential of using (only) interictal data is particularly attractive in a clinical setting.^21,22^ Despite initially promising results from previous studies, open questions remain about, for example, the accuracy/predictive ability of the method, whether it generalizes to all patients regardless of implantation strategy, and whether there are specific timescales or timepoints that are more predictive than others.

We formulated these open questions into three concrete challenges that we will address here. First, functional networks derived from iEEG are always dependent on the spatial location of the electrodes, which differs from patient to patient. Electrodes that are physically closer are also more likely to be highly correlated.^22,23^ Second, the individualized spatial configuration of intracranial electrodes also means that a different number of electrodes are sampling tissue that is ultimately removed or spared by surgery. This makes generalization of network analyses across patients difficult. Third, although iEEG functional networks fluctuate over time,^24,25^ it is not currently known whether these fluctuations affect their ability to predict surgical outcome.

## 2 Materials and Methods

### 2.1 Patients and iEEG and magnetic resonance imaging preprocessing

This retrospective study analyzed data from 55 patients with refractory focal epilepsy from the National Hospital for Neurology and Neurosurgery who had iEEG followed by resection and clinical follow-up of at least 12 months. iEEG data were recorded using a mixture of grid, strip, and stereo-electroencephalography setups. The iEEG data were anonymized and exported, then analyzed under the approval of the Newcastle University Ethics Committee (2225/2017). Patient metadata are shown in Supplementary Information S1.

The iEEG data analyzed consisted of continuous 1-hour segments of interictal EEG sampled at 512 Hz or 1024 Hz that were at least 2 hours away from seizures, as identified by the clinical team. We extracted the 1-hour segment in the afternoon (2-5 PM), where possible, to increase the likelihood of sampling from an awake state. However, it was not possible to retrospectively verify the vigilance state for each segment. Grossly artifactual channels were removed by visual inspection, and all remaining channels were subsequently rereferenced to common average. In accordance with standard practice, each channel was then notch filtered at 50 Hz (infinite impulse response filter with Q factor = 50, 4th order zero phase lag) and bandpass filtered (Butterworth 4th order zero phase lag) between 1 and 70 Hz.

**Figure 1:**
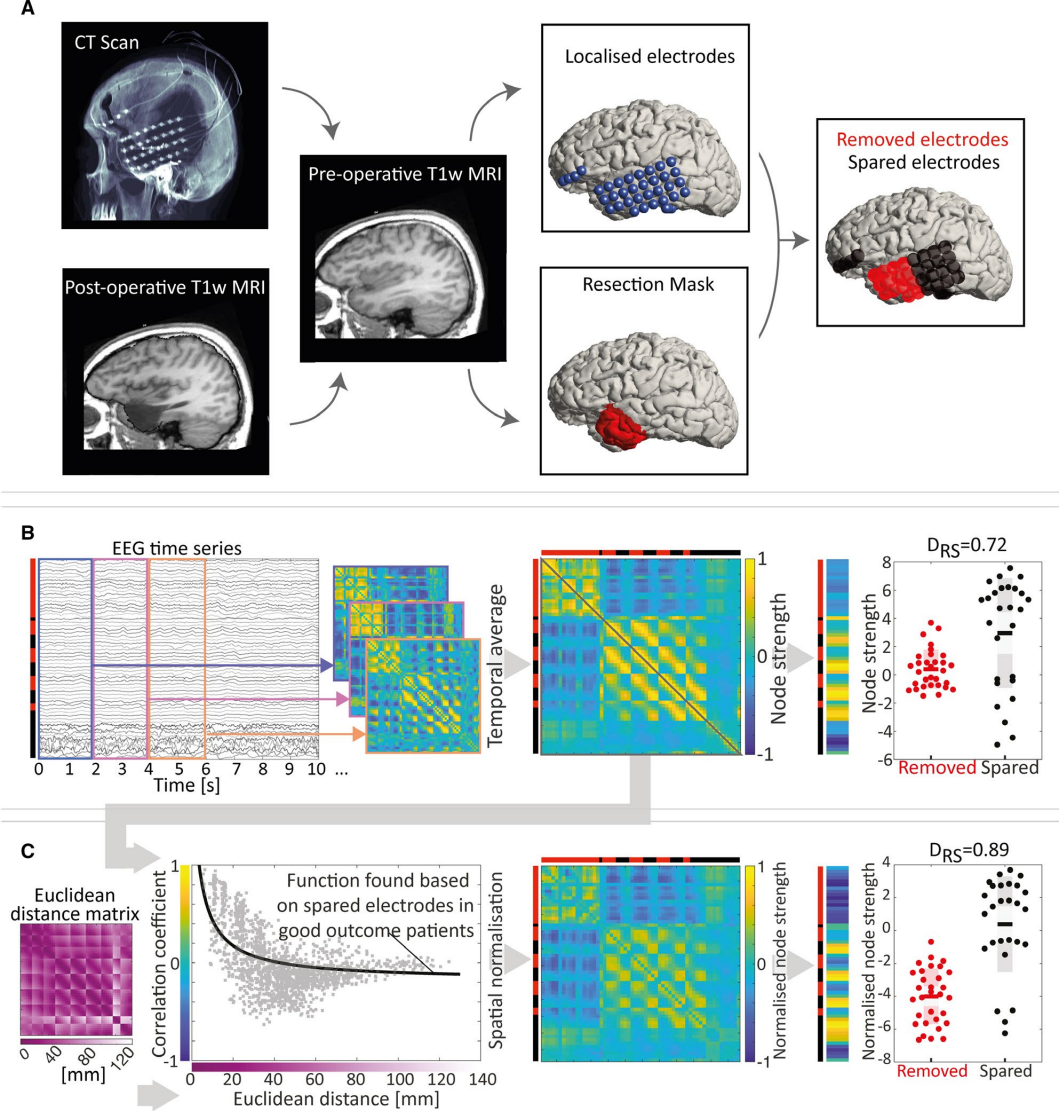
Summary of the processing pipeline. A, Computed tomography (CT) and postoperative T1-weighted (T1w) magnetic resonance imaging (MRI) scans were linearly registered to the preoperative T1w MRI scan. Electrodes, shown in blue, were localized from the registered CT and marked. A mask was additionally manually drawn (shown in red and projected to the cortical surface for visualization) to cover those areas removed by surgery. Electrode contacts located within 5 mm of the volumetric surgery mask were then identified as removed (shown in red), and all others were identified as spared (shown in black). The Euclidean distance between each electrode contact is also calculated in millimetres. B, Two-second nonoverlapping correlation matrices were computed from the electroencephalographic (EEG) time series, and their mean (temporal average) matrix was calculated. By summing the rows of the temporal average matrix, the node strength was calculated. The difference in node strengths for removed and spared electrode contacts was then computed as the D_RS_ measure, with 1 indicating perfect separation of the removed versus spared tissue and 0.5 indicating no separation. C, To derive a spatially normalized temporal average matrix, we applied a spatial regression that was precalculated based on spared electrode contacts from good outcome patients. After applying the regression, we retained the residuals as the spatially normalized temporal average matrix, which allows calculating a normalized node strength and D_RS_ value. The pipeline was applied to each patient

To delineate the iEEG electrode contacts that overlapped with the subsequently surgically removed tissue, we first mapped the spatial position of the iEEG electrodes to the space of the preoperative structural magnetic resonance imaging (MRI) using intraoperative MRI and computed tomography in a semiautomated fashion.^26^ We also manually delineated the surgically removed tissue in the space of the preoperative MRI using rigid-body registration of the postoperative T1-weighted (T1w) MRI to the preoperative T1w MRI.^9^ Any iEEG electrode contact that was within 5 mm of surgically removed tissue was deemed as a “removed electrode contact.” All others were marked as spared electrode contacts. For one patient (patient 851), a postoperative MRI was unavailable and the surgery report from the clinical team was therefore used to identify the resected electrode contacts. The procedure is summarized schematically in Figure 1A.

### 2.2 Functional network derivation and network quantification

Functional brain networks were derived from the 1-hour iEEG segments. We applied Pearson correlation to 2-second sliding windows (without overlap) of the broad band (1-70 Hz) iEEG data and subsequently averaged the correlation matrices over all windows to obtain one functional network matrix per subject.

To quantify the network properties of each node, we used average node strength, which is a measure of the average level of correlation of a node with all other nodes. It has been suggested that this quantity is indicative of epileptogenic tissue, and we show in Supplementary Information S3 that it recapitulates a quantity derived from a dynamical model of epileptogenic tissue we previously suggested.^18^ To quantify whether the node strength (derived based on all electrode contacts) of removed electrode contacts differed from the node strength of the spared electrode contacts, we used the area under the receiver operating characteristic curve (AUC), which is equivalent to the normalized nonparametric Mann-Whitney *U* statistic. We chose this measure because it is based only on the rank order of the node strengths, and thus robust to outliers and nonnormal distributions in node strength. In the following, we will term this measure D_RS_, which stands for the distinguishability of the removed node strengths versus the spared node strengths and has a single value per patient. A D_RS_ value equal to 1(0) indicates all spared electrode contacts have a higher (lower) node strength than all removed electrode contacts. This procedure is summarized schematically in Figure 1B.

Note that we intentionally kept our analysis to the most basic measure of functional connectivity (Pearson correlation) with the most minimal preprocessing. This allowed us to focus on more general challenges that would affect any measure of functional connectivity and their nodal properties (including dynamical model derived nodal properties). It also allows future studies to easily compare to our results as a reference. In the future, ideally with larger sample sizes and multicenter datasets, systematic searches for the optimal functional connectivity measures and models could be performed.

### 2.3 Spatial normalization

That electrode contacts that are closer together in space are more likely to be correlated introduces a bias in the functional networks that depends on each subject’s implantation. We therefore applied a spatial normalization to reduce this bias. We used spared electrode contacts from good outcome (International League Against Epilepsy [ILAE] class 1) patients only, which represents signals from nonepileptogenic tissue as a baseline to establish how correlation coefficients change as a function of spatial (Euclidean) distance. We fitted a rational polynomial (rat11 in MATLAB) to model the decay of electric potentials as a function of space. Once this baseline function was determined, all correlation coefficients in the functional network matrices were normalized by computing the residuals to this baseline function. The resulting spatially normalized correlation coefficients quantify the extent of correlation between two electrode contacts that is beyond what is expected due to their spatial proximity to each other. This procedure is summarized schematically in Figure 1C.

### 2.4 Spatial coverage

Due to clinical need, the spatial coverage of the implanted electrode contacts generally differs from patient to patient. Thus, the spatial sampling of the surgically removed/spared tissue differs in terms of total number of electrode contacts and amount of tissue removed. To account for the variability, we counted the number of removed electrode contacts and the number of spared electrode contacts in each patient. We then successively excluded subjects from our analysis based on the minimum number of removed and spared electrode contacts (n_x_) to observe the effect of spatial coverage on our results. Although we want to account for total sampled volume, we also want to ensure balance between coverage of the spared and resected tissue. For example, at n_x_ = 1, all 55 subjects are included. At n_x_ = 20, only subjects who have at least 20 removed electrode contacts and at least 20 spared electrode contacts are retained for analysis, which in our case consists of 27 subjects. In our analysis, we scan the n_x_ parameter from 1 to 40 and report the results for each value. The rationale behind this measure is that larger n_x_ values can be interpreted as providing a better network representation and sampling of both removed and spared networks. It then follows that if we have captured the network better, our discrimination between outcome groups should improve.

### 2.5 Temporal variability

A longstanding open question in the field is whether the temporal variability of the interictal functional networks^24,25^ affects its ability to delineate epileptogenic tissue.

We first addressed the question of timescale by dividing the 1-hour iEEG segment into several smaller nonoverlapping segments (segments of length = 4 seconds, 10 seconds, 20 seconds, 40 seconds, 1 minute, 3 minutes, 6 minutes, and 10 minutes) and measuring their performance when repeating the same analysis. Specifically, for each segment, we apply a 2-second nonoverlapping window to create an average functional network matrix for this segment. For example, the 40-second segment functional network matrix is created from 20 windows, whereas the 10-second segment is generated from only five windows.

We next investigated the performance of two other separate 1-hour segments from the same subject (at least 2 hours away from seizures, and at least 4 hours away from the other iEEG segments). In some patients, it was not possible to find such a second or third 1-hour segment, leaving 53 subjects with a second segment, and 51 subjects with a third segment).

All analyses were performed independently on each segment; all the steps including spatial regression onward were performed for each segment without knowledge of the other segments.

### 2.6 Statistical analysis of relationship to surgical outcome

To investigate whether D_RS_ contains useful information to explain postsurgical outcomes, we compared D_RS_ between good (ILAE class 1) and poor outcomes (ILAE class 2 and above). We measured the AUC as the main metric, where AUC = 1 shows that D_RS_ can fully distinguish good and poor outcome patients. Conversely, an AUC of 0.5 indicates that D_RS_ cannot distinguish between outcome groups. We also tested for the statistical significance of the AUC by performing the rank sum test between the good and poor outcome patients for the D_RS_ measure. We obtained 95% confidence intervals of the AUC based on a logit transformation.^27^ Supplementary Information S2 shows equivalent results using a cross-validated AUC. Thus, we will use the term “predict” in the following, as it holds both in the loose sense of separating groups, as well as in the strict sense of cross-validated performance as a predictor.

## 3 Results

In this study, we compare network properties (in particular node strength) of interictal iEEG functional networks between the surgically removed and spared tissue to predict surgical outcome (seizure freedom) in individual patients. We will specifically address the following three questions: (1) Does spatial normalization of functional networks increase the ability to distinguish between outcome groups? (2) Does increased coverage of removed and spared tissue lead to increased distinction between outcome groups? and (3) Does the choice of timescale or timepoint affect the ability to distinguish between outcome groups?

### 3.1 Spatial normalization of interictal functional networks improves distinction between outcome groups

**Figure 2:**
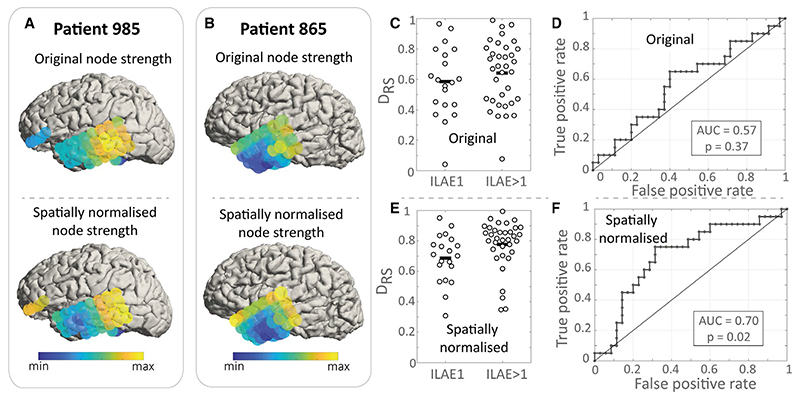
Spatial normalization improves the discrimination between outcome groups. A, B, Node strength before and after spatial normalization for two sample patients. C, International League Against Epilepsy (ILAE) outcome groups show similar D_RS_ values, meaning that the difference in node strength between removed and spared nodes does not explain outcome. Each dot is an individual patient. D, Receiver operating curve for the data presented in C shows poor discrimination between outcome groups. E, After spatial normalization, patient groups show significant differences in their D_RS_ values. F, These can discriminate outcome groups with an area under the receiver operating characteristic curve (AUC) of 0.7

We first investigate whether node strength computed from raw, spatially unnormalized networks discriminates between outcome groups. Figure 2A,B (upper panels) shows the node strength computed for two example patients, which are then used to calculate the D_RS_ value. This single D_RS_ value measures the difference in node strength between removed and spared electrode contacts in an individual. Figure 2C shows the D_RS_ value for all 55 patients in our study, and we find no substantial difference in D_RS_ value between outcome groups (Figure 2D). Given that electrode contacts that are more spatially proximal are more likely to have higher functional connectivity, we next sought to determine whether normalizing for this effect increases discrimination between groups using the D_RS_ measure. To this end, we used a null model for spatial normalization, which accounts for the spatial proximity of electrode contacts (see Materials and Methods). The normalization subsequently impacts the node strength (cf Figure 2A,B upper and lower panels), and in our data, it improves the distinction between outcome groups (compare Figure 2C,D,E,F). For our remaining analysis, we will therefore use spatially normalized functional networks.

### 3.2 Increased coverage of removed and spared tissue improves distinction between outcome groups

**Figure 3:**
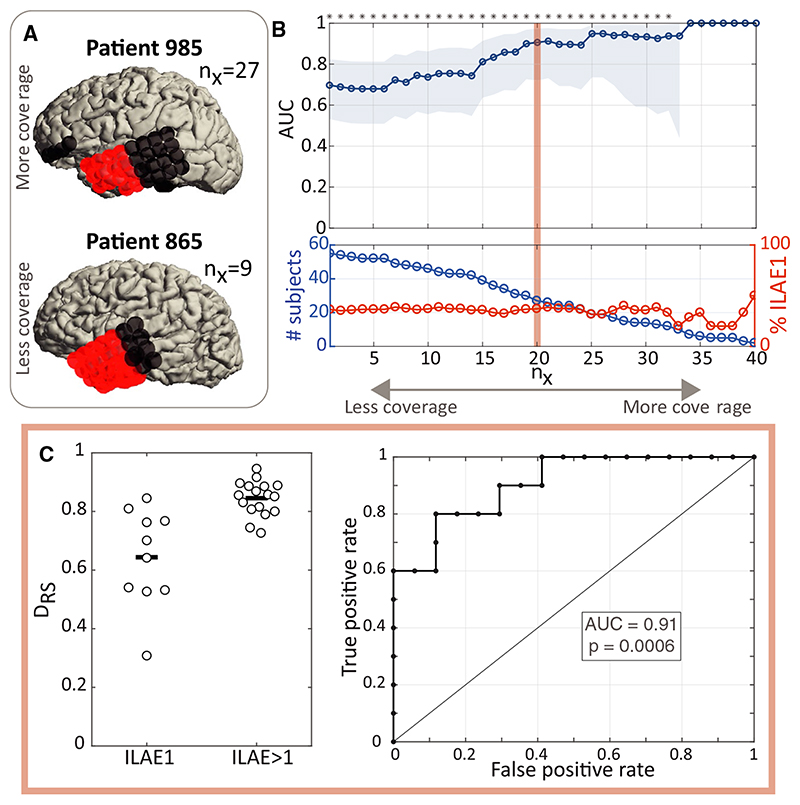
Greater coverage of spared and removed networks is associated with greater discrimination between outcome groups. A, Removed (red) and spared (black) electrode contacts are shown for two sample patients. Patient 985 has greater sampling of spared tissue (27 electrode contacts) than patient 865 (nine electrode contacts). B, Top: Scanning area under the receiver operating characteristic curve (AUC; blue line) over n_x_, where at each n_x_ value, only patients with at least n_x_ electrode contacts in removed and spared tissue are included in the analysis. Shaded blue area indicates the 95% confidence interval for the AUC. For high n_x_ too few subjects remained for analysis to obtain a confidence interval. Bottom: The number of patients included (blue line) and the percentage of good outcome (red line) patients for each n_x_ value. C, At a value of n_x_ = 20, D_RS_ values between outcome groups, and receiver operating characteristic curve are shown. ILAE, International League Against Epilepsy

Spatially undersampling networks can directly lead to changes in the estimated network properties,^28^ and thus we investigated the impact of spatial sampling on our ability to distinguish outcome groups. Figure 3A shows two example patients: one has a large number of electrode contacts located in both removed and spared tissue, but the other one only has nine electrode contacts located in the spared tissue. Therefore, these two patients are not directly comparable in terms of the network properties of their spared tissue. To account for this issue, we successively excluded patients with a low n_x_, which is their minimum number of electrode contacts in both spared and removed tissue. As we increase coverage of removed and spared tissue (n_x_), the distinction between outcome groups in terms of AUC values becomes clearer (Figure 3B). For example, at n_x_ = 20 (we only use the subset of patients who have at least 20 electrode contacts in spared and in removed tissue), 27 patients remain for the analysis, and we find outcome class is distinguishable with an AUC = 0.91 (Figure 3C). Note also that the proportion of good versus poor outcome patients does not change substantially over n_x_ (Figure 3B bottom panel red line). Cross-validated AUCs follow a similar trend of increasing AUC for greater coverage (Supplementary Information S2).

### 3.3 Interictal functional networks fluctuate over time, but these fluctuations do not affect the distinction between outcome groups

**Figure 4:**
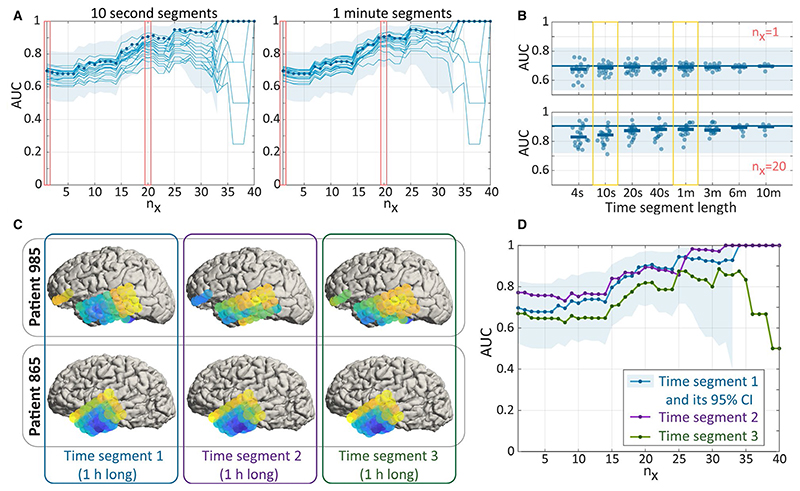
Consistent results for networks sampled at different times. A, Left: Areas under the receiver operating characteristic curve (AUCs) for 20 separate 10-second segments are shown as individual thin blue lines. AUC of the 1-hour segment (thick blue line) and 95% confidence intervals (CIs; shaded blue area) are reproduced from Figure 3B for reference. Right: The same for 20 separate 1-minute segments. B, AUC for segments of different length shown for n_x_ = 1 and n_x_ = 20. The horizontal blue line (shaded area) indicates the AUC (CI) for the 1-hour segment for reference. Thick horizontal blue bars indicate the mean AUC of all segments of a particular length. C, Node strength for two sample patients for three separate 1-hour time segments. Time segment 1 panels are identical to those in the lower panels of Figure 2A,B. D, Blue line and shaded area are from the first 1-hour segment and reproduced from Figure 3B for reference. Purple and green lines indicate the AUC for the second and third 1-hour segments, respectively. Note that all functional networks of all segments were derived by averaging over correlation matrices from 2-second nonoverlapping windows in this figure; i.e, the window size to obtain correlations of the time series stayed constant in all segments

For practical applications using interictal iEEG functional networks to delineate epileptogenic tissue, it is important to understand whether and how our results change if different underlying data are used. Specifically, we investigate whether networks generated from segments of different duration and from different timepoints affect our main findings. We systematically scanned segments of shorter durations and measured their ability to distinguish outcome groups in our cohort (Figure 4A,B). Typically, a 10-second segment does not perform significantly worse than a 1-hour segment over all n_x_. For all segments of all lengths, their AUCs lie largely within the 95% confidence interval of the AUC for the 1-hour segment (Figure 4A,B). We do, however, note that the AUC varies more from segment to segment for shorter segments (≤10 seconds), indicating that consistency of results may drop for short segments. To test whether a different 1-hour time segment would change our AUCs substantially, we repeated the same analysis for two different 1-hour time segments that are at least 4 hours away from any other segment in each patient. Figure 4C shows the node strength of two sample subjects over all three 1-hour segments, and although there are some variations, the gross spatial pattern remains stable. We quantified the between-segment similarity in terms of node strength, and the average correlation between segments across subjects was *c* = 0.946. This consistency is also reflected in the AUCs distinguishing outcome groups (Figure 4D), where the two new segments lie within the confidence interval of the original segment.

## 4 Discussion

Interictal iEEG network-based approaches to predict seizure freedom after surgery and to identify epileptogenic tissue have attracted interest in recent years. However, major issues regarding spatial bias, incomplete coverage, and temporal stability have remained relatively unexplored. Our study makes three key contributions in this regard. First, we found that spatial normalization substantially increases the discrimination between outcome groups. Second, we found that increased coverage of removed and spared networks was associated with greater discrimination between outcome groups, but not necessarily better outcomes. Third, our results are in agreement for a wide range of timescales, from minutes up to hours. Our work confirms that interictal iEEG network analysis holds value for predicting seizure freedom after surgery, but also highlights challenges in the practical use of this method.

The first challenge is that of normalization. Specific functional network patterns in healthy subjects underpin normal brain function. When observing such networks in patients with epilepsy, the pathological patterns should be distinguished from the healthy ones. However, in most iEEG-based functional network studies, this distinction is not made. The need to establish baselines derived from healthy tissue for iEEG has nevertheless also been recognized by others.^23,29^ In our work here, we used spatial normalization of functional networks as a natural way to measure signal similarity relative to a baseline, where the baseline is derived based on spared electrode contacts in good outcome patients. The spatial normalization procedure itself could be improved in the future, with a clearer understanding of what biophysical but also biological factors (eg, lobe- or region-specific functions) should be accounted for. Besides spatial information, white matter pathways and shared gene expression between regions also explain functional relationships.^23^ In the future, these and other variables could also be included to normalize functional networks in iEEG to enhance the detection of the pathological aspects.

The second and most pertinent challenge we highlighted is that of spatial sampling. iEEG only samples specific subnetworks in the brain, which can vary widely between patients. It is clear that such subnetworks do not necessarily have the same properties as the whole-brain network. Recent analyses demonstrated that even leaving out one node from iEEG functional networks can dramatically change their network properties.^28^ The implication is that the characteristics of the epileptogenic tissue/network may change depending on the subnetwork sampled, which may explain some conflicting results in the literature.^30^ Thus, the restricted spatial sampling inherent in iEEG is a natural limitation in the context of functional networks, and we showed that it directly impacts upon how informative the functional networks are for distinguishing outcome groups. Other studies using different patient cohorts (with different implantation strategies) may therefore achieve better or worse AUCs as a direct consequence of the coverage of the patients in the study. In other words, interictal iEEG functional network approaches may completely fail to predict surgical outcome, if the spatial sampling is very sparse. Finally, it is important to note that in our study, increased coverage (higher n_x_) was associated with increased discrimination between outcome groups, but was not necessarily associated with better outcomes. Future studies combining scalp EEG or other recording modalities with iEEG may reveal how the iEEG subnetwork can be related to whole-brain networks to improve localization.

The third challenge we addressed is that of temporal scale in the analysis of iEEG interictal functional networks. Temporal fluctuations of iEEG functional networks are well studied during epileptic seizures and the preictal periods.^11,31,32^ However, the interictal iEEG functional networks are often treated as static,^15,18,20^ and in contrast to the ictal networks, it is suggested that interictal networks are stable over time,^33,34^ or at least that the pathological component is persistent through time.^15,35^ Rather than determining stability or fluctuations of interictal functional networks as such, we asked the simpler question of whether the timescale or timepoint matters for discriminating between outcome groups. In our cohort, the timescale and timepoint did not dramatically impact our results. However, this result should not be directly interpreted as evidence for stability of the interictal functional networks. Future work should investigate what aspects of in-terictal iEEG are variable/static, which may also highlight the causal link between interictal EEG and epileptogenic tissue. Relatedly, future work should also study whether brain states or vigilance states affect the predictive value of these functional networks.

In terms of practical application of this method, we only presented results on the prediction of postsurgical outcome based on preoperative iEEG data. Similar to many other studies (see particularly Figure 8 in Taylor et al^9^), this can directly serve as an in silico tool to delineate the epileptogenic tissue. As the data needed for prediction are purely preoperative, it means that many surgery strategies can be tested in silico, and each strategy associated with a predicted outcome. This approach can also be used to design the most minimal resection required. We contend that any algorithm that can predict surgical outcome based on preoperative data only is also at the same time an in silico tool to delineate the epileptogenic tissue. In the future, we envisage algorithms combining different preoperative data modalities, and using clear normative baselines for each modality, to delineate the optimal tissue to remove for postoperative seizure freedom.

Taken together, our results support the use of interictal iEEG networks for predicting surgical outcome and provide considerations and practical solutions for its clinical use. Future studies should investigate the generalizability of the approach across multiple clinical sites and assess the combined use with other noninvasive whole-brain modalities. The principles investigated here may also serve as an inspiration for the investigation of other neurological disorders.

## Supplementary Material

Additional supporting information may be found online in the Supporting Information section.

Supplementary material

Supplementary table

## Data Availability

Functional network matrices and analysis code are available at the following: https://doi.org/10.5281/zenodo.3837441.

## Key Points

- Interictal functional networks were generated using intracranial EEG for 55 patients
- Predictions of surgical outcome were made and validated against actual outcomes
- Accounting for spatial proximity of electrodes improves prediction accuracy
- Accounting for number of electrodes allows excellent prediction accuracy (area under the receiver operating characteristic curve > 0.9)
