# Supplementary material for: Interictal intracranial electroencephalography for predicting surgical success: The importance of space and time
Source: Epilepsia. Author manuscript; Available in PMC 2021 Jul 9. (PMC7611164; doi:10.1111/epi.16580)
Supplement: Supplementary material [file EMS129523-supplement-Supplementary_material.docx]

**Supplementary material**

**Supplementary material S1**

Table of patient data.

**Supplementary material S2: Cross validated analysis gives similar results**

The results reported in the main text describe the distinguishability of the two outcome groups - i.e. how different they are. To test the generalisability of the approach for prediction on unseen data, we adopted a leave-one-out cross validation approach described here. We used the D_RS_ value as a single input feature and a support vector machine (SVM) classification model built for all but one of the patients. This training model finds an optimal D_RS value that separates the outcome groups. The remaining (test) patient is then input to the model and a probability of class membership calculated. This process is repeated for all subjects using the fitcsvm command in MATLAB, resulting in a score for each run/subject. To reduce bias with respect to imbalanced data (an uneven number of good versus poor outcomes), we used a prior probability of 0.5 (i.e. equal for each class). The figure below shows the cross-validated AUC for this approach, which confirms the same trend of increased AUC with increasing coverage (n_x_) (compare with main text figure 3).


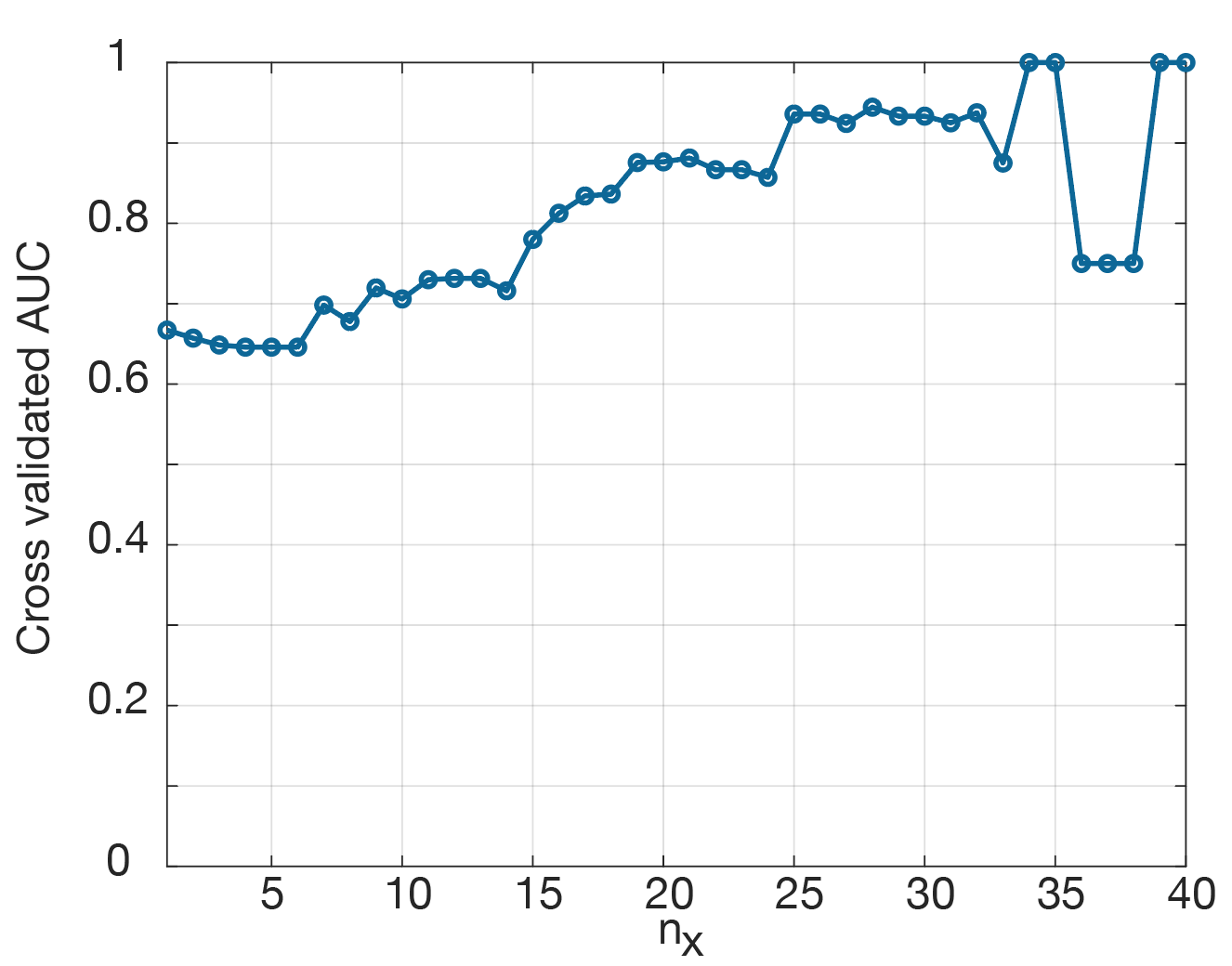


**Supplementary material S3: Model simulations perform similarly to node strength.**

In our previous work (Sinha et al, 2017), we showed that escape times computed from model simulations were useful for predicting outcomes after epilepsy surgery. In the main text of this manuscript we use a simpler measure - the node strength - for our analysis. Here we show that the node strength and escape times perform similarly in terms of predicting surgical outcome.

For model simulations we used the same model as in our previous study. In brief, individual nodes are placed in a bistable parameter region with the potential to transition between one state (a fixed point) and another (an oscillation). Transitions are driven by time varying noise input to individual nodes and input from other nodes. Input from other nodes is parameterised from the patient-specific functional connectivity matrix. Since the spatial regression procedure introduces negative values into the connectivity matrix, in this study we normalise the connectivity matrix by the minimum and maximum value such that they equal 0 and 1, respectively, before model simulation. To compute escape times, all nodes are initially set to zero, and the first occurrence of a high amplitude oscillation is recorded. As in Sinha et al (2017), this simulation is repeated 1000 times with different noise seeds, and the mean escape time across the 1000 runs is used for each channel. Note that the normalisation choice is a linear transformation of the connectivity, and ensures that all patients have a comparable escape time. However, it does not alter the relative proportions of escape time in each channel and patient.

The figure below (panel A) shows that the D_RS_ values, when computed using the model, are highly correlated with the D_RS_ values calculated when using the simpler measure of node strength. It then follows that the other results reported in the main manuscript are also highly similar when using the computational model (panels B,C and D). Note A & B are obtained with all the patients (i.e. n_x_=1).

**
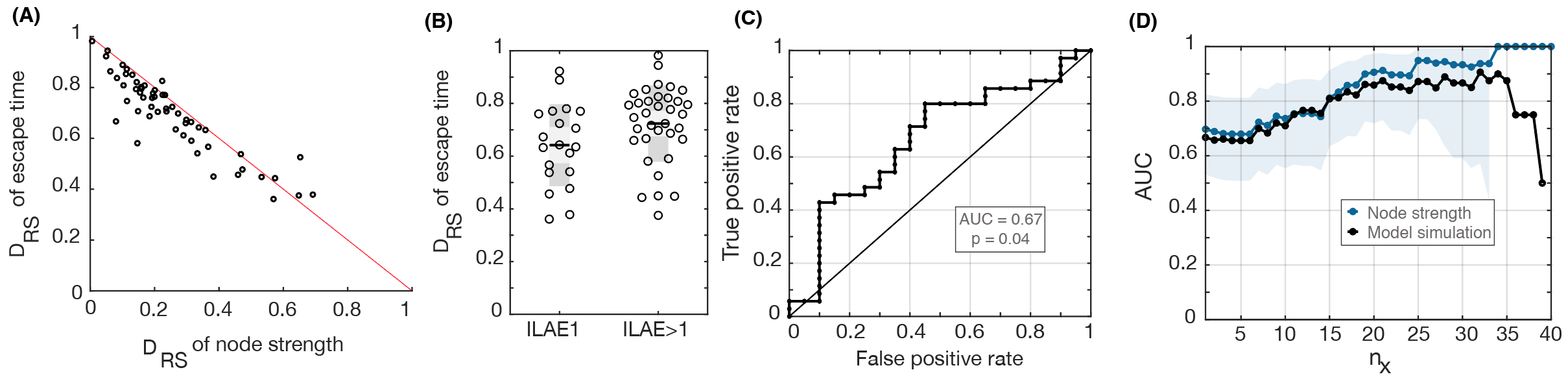
**

Although there are benefits to the use of node strength as a simple measure for predicting patient outcomes, including speed of calculation and ease of interpretation there are drawbacks which a modelling approach can overcome. For example, node strength only reflects the influence of connectivity, it cannot readily implement additional information, such as localised properties of the node (e.g. local atrophy which can measured by MRI). Furthermore, signal propagation delays via white matter tracts – which may also be altered in patients - are also not directly included. Computational modelling has been used to overcome both of these technical limitations with the intention of utilising multimodal information for prediction (Proix et al 2017, Hutchings et al 2015).
