# Supplementary material for: Interictal intracranial electroencephalography for predicting surgical success: The importance of space and time
Source: Epilepsia. Author manuscript; Available in PMC 2021 Jul 9. (PMC7611164; doi:10.1111/epi.16580)
Supplement: Supplementary table [file EMS129523-supplement-Supplementary_table.pdf]

Table S1

Patient ID	n_x	ILAE Class	D_{RS}	Total num. contacts	Num. depth contacts	Num. subdural contacts	Operation Type	Operation Side
592	16	3	0.799	59	59	0	T Lx	R
687	2	1	0.633	17	17	0	T Lx	L
770	34	3	0.871	74	12	62	P Lesx	L
782	20	1	0.710	54	12	42	T Lx	L
800	28	5	0.804	62	12	50	T Lx	L
803	18	4	0.841	53	53	0	T Lx	R
815	18	5	0.843	40	12	28	T Lx	L
821	15	4	0.776	46	30	16	T Lx	L
851	4	5	0.805	68	0	68	F Lx	L
865	9	1	0.899	52	18	34	T Lx	L
873	35	4	0.858	72	12	60	T Lx	L
874	15	5	0.688	69	8	61	F Lx	L
902	25	1	0.846	63	12	51	T Lx	L
909	8	1	0.438	26	26	0	T Lx	L
910	45	4	0.945	120	0	120	F Lx	L
931	22	4	0.886	54	8	46	T Lx	L
940	20	5	0.938	72	12	60	T Lx	R
943	14	1	0.667	32	26	6	T Lx	R
965	27	3	0.859	90	6	84	P Lesx	R
985	27	4	0.888	58	18	40	T Lx	L
998	7	4	0.667	22	22	0	T Lx	L
999	33	1	0.771	77	8	69	F Lx	L
1005	7	4	0.333	88	16	72	T Lx	R
1006	32	1	0.768	104	0	104	F Lx	R
1038	3	4	0.920	28	28	0	T Lx	R
1055	18	4	0.775	38	38	0	T Lx	R
1064	40	4	0.855	97	18	79	T Lx	L
1074	12	1	0.775	32	32	0	T Lx	L
1085	15	1	0.951	84	8	76	F Lx	L
1096	28	2	0.820	84	84	0	F Lx	R
1106	39	4	0.831	122	0	122	F Lx	R
1109	25	1	0.541	54	54	0	T Lx	R
1111	33	1	0.531	102	12	90	T Lx	R
1119	36	1	0.706	124	12	112	F Lx	L
1163	42	1	0.811	109	0	109	F Lx	L
1167	20	4	0.708	51	10	41	P Lx	L
1168	34	2	0.725	94	12	82	F Lx	L
1178	21	4	0.789	82	0	82	F Lx	L
1179	25	4	0.746	57	18	39	T Lx	L
1182	31	3	0.915	76	16	60	P Lesx	R
1196	10	3	0.994	27	27	0	T Lx	R
1200	17	1	0.669	73	73	0	T Lx	R
1211	17	4	0.467	74	18	56	T Lx	R
1216	24	4	0.811	74	74	0	O P Lx	L
1220	39	5	0.896	96	14	82	T Lx	L
1223	7	3	0.619	82	22	60	F Lx	L
1258	29	1	0.532	71	12	59	T Lx	R

Patient ID	n_x	ILAE Class	DRS	Total num. contacts	Num. depth contacts	Num. subdural contacts	Operation Type	Operation Side
1275	11	4	0.364	50	50	0	T O Lesx	R
1284	19	3	0.436	64	64	0	F Lx	R
1344	22	1	0.312	59	59	0	T Lx	R
1379	11	3	0.858	78	78	0	T Lx	R
1382	16	1	0.730	84	84	0	T Lx	R
1389	34	3	0.888	78	78	0	F Lx	L
1395	24	1	0.636	95	95	0	T Lx	L
1397	16	1	0.831	77	77	0	T Lx	R

Abbreviations: Lx Lobectomy, Lesx Lesionectomy, T Temporal, F Frontal, O Occipital, P Parietal, L Left, R Right
